# Уровни инсулина, гликемии, резистентности к инсулину и функциональной активности β-клетки при различном образе жизни коренного жителя Арктики. Есть ли предпосылки к развитию сахарного диабета и какого?

**DOI:** 10.14341/probl13411

**Published:** 2024-11-05

**Authors:** А. В. Стрелкова, Ф. А. Бичкаева, О. С. Власова, Е. В. Нестерова, Б. А. Шенгоф, Т. Б. Грецкая

**Affiliations:** Федеральный исследовательский центр комплексного изучения Арктики им. акад. Н.П. Лаверова УрО РАН; Федеральный исследовательский центр комплексного изучения Арктики им. акад. Н.П. Лаверова УрО РАН; Федеральный исследовательский центр комплексного изучения Арктики им. акад. Н.П. Лаверова УрО РАН; Федеральный исследовательский центр комплексного изучения Арктики им. акад. Н.П. Лаверова УрО РАН; Федеральный исследовательский центр комплексного изучения Арктики им. акад. Н.П. Лаверова УрО РАН; Федеральный исследовательский центр комплексного изучения Арктики им. акад. Н.П. Лаверова УрО РАН

**Keywords:** инсулин, глюкоза, HOMA-IR, HOMA1-%β, коренные жители Арктики

## Abstract

**ОБОСНОВАНИЕ:**

ОБОСНОВАНИЕ. Современные исследования дают основания полагать, что смена уклада жизни аборигенов Арктики приводит к утрате «адаптивного полярного метаболического типа», характеризующегося интенсификацией белкового, оптимизацией липидного и минимизацией углеводного обменов при низких концентрациях инсулина. Что удается отстоять в эпоху перемен?

**ЦЕЛЬ:**

ЦЕЛЬ. Оценить уровень инсулина, гликемии, инсулинорезистентности и секреторной активности β-клетки у аборигенов Арктики относительно их образа жизни.

**МАТЕРИАЛЫ И МЕТОДЫ:**

МАТЕРИАЛЫ И МЕТОДЫ. Проведено поперечное исследование популяции аборигенов Арктики (ненцы, коми) от 22 до 60 лет. В сыворотке крови методом ИФА исследованы уровни инсулина, спектрофотометрическим — глюкозы. Рассчитаны индексы HOMA-IR и HOMA1-%β.

**РЕЗУЛЬТАТЫ:**

РЕЗУЛЬТАТЫ. Обследовано 397 человек; 89 чел. (22%) — кочующих аборигенов (КА), 44 (50%) муж.; 308 чел. (78%) — оседлых (ОА), 69 (22 %) муж. Уровень инсулина — 6,0 [3,5–11,8] мкЕд/мл — был значимо ниже у КА в сравнении с ОА — 8,3 [4,6–13,1] мкЕд/мл, р=0,006. Гликемия 4,6 [4,2–5,0] и 4,6 [4,1–5,2] ммоль/л — без различий по образу жизни. Величина HOMA-IR у КА — 1,3 [0,7–2,4] значимо ниже, чем у ОА 1,8 [0,95–2,8] усл.ед., р=0,013. Скорректированное (пол, возраст, ИМТ) ОШ наличия IR-HOMA>2 усл.ед. у ОА в 1,8 раза выше, чем у КА, ОШ=0,56; 95% ДИ: 0,33–0,96, р=0,034. Me [IQR] HOMA%β 128 [67–241] и 144 [93–236]% без значимых различий между группами. Случаев «HOMA1-%β<48,9» больше у КА: 17% против 5% у ОА, p<0,001. Скорректированные шансы наличия «HOMA1-%β<48,9» у КА в 3,5 раза выше, чем у ОА; 95% ДИ: 1,56–7,92, р=0,002. Выявлено 56 случаев гликемии ≥5,6 ммоль/л: 13,5% у КА и 14% у ОА; соотношение IR-HOMA, усл.ед /HOMA1-%β/ИМТ кг/м2 составило: 1,8/45/25,2 у КА и 3,0 /88/29,6 у ОА, p<0,001.

**ЗАКЛЮЧЕНИЕ:**

ЗАКЛЮЧЕНИЕ. Сохранение кочевого образа жизни способствует удержанию более низкой концентрации инсулина; при этом уровни гликемии в группах сходные. У КА это обусловлено высокой долей лиц с низкой секреторной активностью β-клетки, преимущественно мужчин; ОА — более инсулинорезистентны. Анализ случаев гликемии ≥5,6 ммоль/л подтвердил генез гипергликемии у КА, связанный с гипофункцией β-клетки с отсутствием случаев ожирения при этом; у ОА — связанный с ростом резистентности к инсулину и ожирением.

## Abstract (en)

**BACKGROUND:**

BACKGROUND: Modern studies suggest that lifestyle changes of the indigenous Arctic residents lead to the loss of the “adaptive polar type of metabolism”, which is characterized by the intensification of protein metabolism, optimization of lipid metabolism, and minimization of carbohydrate metabolism at low insulin concentrations. How to survive the era of change?

**AIM:**

AIM: To assess insulinemia, glycemia, β-cell secretory activity, and insulin sensitivity in Arctic indigenous people in relation to their lifestyle.

**MATERIALS AND METHODS:**

MATERIALS AND METHODS: A cross-sectional study of a population of indigenous Arctic residents (Nenets, Komi) aged from 22 to 60 years was conducted. Insulin levels were studied in blood serum using ELISA, and glucose levels using the spectrophotometric method. Insulin resistance (HOMA-IR) and β-cell function (HOMA1-%β) indices were calculated.

**RESULTS:**

RESULTS: 397 people were examined; 89 (22%) of them were nomadic people (NP) and 44 (49%) were male. Another 308 (78%) were sedentary people (SP), and 69 (22%) were male. The insulin level was significantly lower in NP (6.0 [3.5–11.8] µU/ml) compared to SP (8.3 [4.6–13.1] µU/ml), p=0.006. There was no difference in glycemia (4.6 [4.2–5.0] in NP and 4.6 [4.1–5.2] in SP) between lifestyles.

The HOMA-IR was significantly lower in NP (1.3 [0.7–2.4]) than in SP (1.8 [0.95–2.8]), p=0.013. IR-HOMA >2 units was 1.8 times more frequent in the SP than in NP, with adjusted for sex, age, and BMI OR=0.56; 95% CI: 0.33–0.96, p=0.034. The median HOMA1-%β was 128 [67–241] % in NP and 144 [93–236]% in SP with no significant differences between groups. The proportion of individuals with HOMA1-%β <48.9 was 17% in NP versus 5% in SP, p<0.001. The adjusted odds of having HOMA1-%β <48.9 in NP are 3.5 times higher than in SP; 95% CI: 1.56–7.92, p=0.002. Fifty-six cases of glycemia ≥5.6 mmol/l were identified: 13.5% in NP and 14.3% in SP. The ratio IR-HOMA >2/ HOMA1-%β <48.9/BMI was 1.8 units/45%/25.2 kg/m2 in NP and 3.0 units/88%/29.6 kg/m2 in SP.

**CONCLUSION:**

CONCLUSION: Maintaining a nomadic lifestyle helps keep lower insulin concentrations; at the same time, glycemic levels in the groups were similar. In the NP group, there was a high proportion of individuals with low β-cell secretory activity, predominantly men; in the SP group, more individuals were insulin-resistant. Analysis of cases of glycemia ≥5.6 mmol/l confirmed, that hyperglycemia in a nomadic lifestyle was associated with β-cell hypofunction and the absence of obesity; on the contrary, in a sedentary lifestyle, it was associated with increased insulin resistance and obesity.

## Обоснование

Метаболическая «пандемия», активно распространяющаяся по всему миру [1][2], ассоциированная с развитием гиперинсулинемии, инсулинорезистентности, сопряженная с ожирением, дислипидемией, воспалением, стрессом эндоплазматического ретикулума, оксидативным и карбонильным стрессом [3], долгое время не затрагивала коренных жителей Арктики [4][5]. Этот феномен объяснялся способностью переключения энергетического обмена с «углеводного» типа на «жировой» и лег в основу концепции «полярного метаболического типа» [6]. Считается, что такая перестройка метаболизма лишь частично генетически детерминирована [7][8] и сформировалась в процессе онтогенеза под влиянием традиционного кочевого уклада жизни и преимущественно белково-липидного рациона питания, обеспечив, при «бережливом генотипе» (Neel, 1964 г.), возможность увеличения основного обмена на 10–33% по сравнению с жителями умеренных широт [8][9] и крайне низкую распространенность в популяции ожирения, сахарного диабета (СД) и сердечно-сосудистой патологии [4][5][7][9].

Одной из ключевых составляющих данной концепции была возможность поддержания сбалансированного энергетического баланса посредствам повышения уровня кортизола и удержания инсулина (Инс) в низких значениях референсного диапазона при сохранении популяционной тенденции к низко-нормальным уровням гликемии [6][10].

С середины прошлого века, на протяжении жизни 3–4 поколений (чрезвычайно короткий в эволюционном плане период), значительная часть коренных северян оказалась перед необходимостью адаптироваться к оседлому образу жизни и пищевому рациону, значительно отличающемуся от традиционного. Одно из основных изменений диеты связано с резким увеличением доли углеводов, потребление которых сравнялось со средними показателями по РФ (40 кг на человека в год) и стало больше, чем в Европе (36,2 кг). Только потребление сахарозы коренными жителями Арктики (как кочевниками, так и оседлыми) возросло в 2 раза (с 30 до 65 г/сут.) [8].

Произошедшие перемены обозначились на современном этапе увеличением случаев ожирения, артериальной гипертензии и сахарного диабета [4][5][8].

Полученные нами данные (2009–2018 гг.) свидетельствуют о повышении средних значений гликемии у жителей Европейской Арктики РФ [11] относительно показателей конца прошлого века [10] и созвучны с трендами эпидемиологических исследований [12][13] и Федерального регистра СД, демонстрирующих рост случаев интолерантности к глюкозе и преимущественно СД 2 типа (СД2), в том числе у жителей северных регионов [4].

В то же время анализ Федерального регистра продемонстрировал интересную особенность: «географический градиент» с более высокой распространенностью СД 1 типа (СД1) на северо-западе страны по сравнению с юго-востоком. Этот перепад сохранился и в сугубо Арктических регионах: так, в Архангельской области и Ненецком автономном округе заболеваемость СД1 — 200–240 на 100 тыс. населения, т.е. в 1,5–2 раза выше по сравнению с Якутией, Камчатским краем и Чукотским автономным округом (100–180 на 100 тыс. населения) [4].

Учитывая вышеизложенное, представляет интерес изучение параметров резистентности к инсулину совместно с показателями секреторной активности β-клетки в популяции коренных жителей Арктики РФ, еще в середине прошлого века практически не страдавших сахарным диабетом и ожирением [4-6].

Инструментами для определения инсулинорезистентности (IR) и функции β-клетки (Fβ) в популяционных исследованиях являются суррогатные индексы, валидизированные с методикой клэмпа, использующие в расчетах как правило постабсорбционные показатели гликемии и инсулинемии [14][15]. Совместная оценка инсулинорезистентности и секреторной активности β-клетки (Fβ) считается наиболее корректной [16] и не встретилась в доступной нам литературе, посвященной изучению гормональной регуляции метаболизма в популяциях аборигенов Арктики.

## ЦЕЛЬ ИССЛЕДОВАНИЯ

Оценка инсулинемии, гликемии, параметров секреторной активности β-клетки и чувствительности к инсулину у коренных жителей Арктики относительно их образа жизни.

## МАТЕРИАЛЫ И МЕТОДЫ

## Место и время проведения исследования

Место проведения. Сбор данных осуществлен экспедициями ФГБУН ФИЦКИА УрО РАН Министерства науки и высшего образования Российской Федерации, г. Архангельск на территориях Арктической зоны Российской Федерации в Ненецком автономном округе (НАО, пос. Нельмин-Нос, Несь), Мезенском районе Архангельской области (пос. Совполье, Сояна, Долгощелье) и Ямало-Ненецком автономном округе (ЯНАО, пос. Сейяха, Тазовский, Гыда, Ныда, Нори, Антипаюта).

Время исследования. С февраля 2009 по апрель 2018 гг. в весенне-зимний период (декабрь–апрель).

## Изучаемые популяции (одна или несколько)

Изучалась популяция коренных жителей Крайнего Севера.

Критерии включения: аборигены НАО, Мезенского района АО и ЯНАО в возрасте 22–60 лет, идентифицирующие себя, согласно анкетным данным, как ненцы и коми не менее чем в двух поколениях, не имеющие на момент проведения исследования жалоб со стороны здоровья, относящиеся к .I–III группе здоровья [17], подписавшие информированное согласие.

Критерии исключения: наличие критериев IV–V группы здоровья [17], а также лица, имеющие в анамнезе диагноз СД и иные эндокринопатии (кроме ожирения) и/или состоящие на диспансерном учете у кардиолога или эндокринолога, имеющие на момент исследования обострение любой хронической патологии, острые заболевания и беременность.

Далее были сформированы 2 группы по образу жизни (ОЖ): первая — кочующие аборигены оленеводы с традиционным кочевым укладом жизни, обследованные в период стоянок вблизи поселков (КА); вторая — оседлые (поселковые) аборигены (ОА).

## Способ формирования выборки из изучаемой популяции (или нескольких выборок из нескольких изучаемых популяций)

Представлена произвольная выборка с распределением по группам в зависимости от образа жизни. Респонденты отобраны случайным образом и составили 2,2% от численности аналогичной по возрасту популяции коренного оседлого (поселкового) и кочующего населения этих территорий (согласно Всероссийской переписи 2010 г.).

## Дизайн исследования

Проведено поперечное исследование. Жители населенных пунктов и кочевники в период стоянок у этих населенных пунктов были заранее проинформированы о предстоящем обследовании; прибывали на фельдшерский пункт в назначенный для обследования день утром, натощак. Все участники дали письменное согласие на участие в исследовании. Затем врачами проводился сбор анамнеза и физикальный осмотр, на основании заключения делался вывод о состоянии здоровья и соответствии критериям включения/исключения для участия в исследовании. Далее проводилось анкетирование по специально разработанной анкете и сбор биологического материала.

## Методы

Всем участникам исследования было выполнено антропометрическое исследование с расчетом индекса массы тела (ИМТ) по формуле Кетле: ИМТ (кг/м2) = вес, кг/рост, м2 и последующей оценкой полученной величины по критериям ВОЗ (1997 г.).

Сбор биологического материала проводился с 08:00 до 10:00 часов строго натощак в вакутайнеры «Beckton Dickinson BP». Полученные образцы центрифугировались, сыворотка или плазма отбиралась в эппендорфы, маркировалась, подвергалась глубокой заморозке и транспортировалась в лабораторию. Иммуноферментным методом с помощью наборов «DRG Instruments Gmb H» на планшетном анализаторе для ИФА (ELISYS Uno, Human Gmb H, Германия) и фотометре StatFax 303 (США) в сыворотке крови определяли содержание инсулина (Инс, референсное значение 2,1–25 мкЕд/мл). Уровень глюкозы (Глю, референсное значение 3,9–6,1 ммоль/л) определяли спектрофотометрическим методом наборами «Chronolab AG».

Рассчитывали индексы:

-HOMA-IR (HOmeostatic Model Assessment for Insulin Resistance) оценки инсулинорезистентности по формуле HOMA-IR = Глю [ммоль/л] * Инс [мкЕд/мл] ÷ 22,5. Референсное значение — 2,0 усл.ед., результат выше предполагал наличие инсулинорезистентности [14][15];

-HOMA1-%β — показатель оценки секреторной функции β-клеток (Fβ) по формулеHOMA1-%β = 20 * Инс [мкЕд/мл] ÷ (Глю [ммоль/л] - 3,5). Снижение показателя являлось предиктором диабета и/или нарушения толерантности к глюкозе. Индекс рассчитывался при значениях глюкозы>3,5 ммоль/л. Референсное значение 48,9 усл.ед.; результат ниже этого предполагал снижение функции β-клеток (дефицит инсулина) [16].

## Статистический анализ

Статистический анализ проводился с использованием программы Statistica (v. 10.0, StatSoft, USA) и StatTech (v. 3.1.8, ООО «Статтех», Россия). Количественные показатели оценивались на предмет соответствия нормальному распределению с помощью критерия Колмогорова. В связи с отсутствием нормального распределения у подавляющего большинства показателей количественные данные описывались с помощью медианы (Me) и нижнего и верхнего квартилей (Q1–Q3). Категориальные данные описывались с указанием абсолютных значений и процентных долей с 95%ДИ. Сравнение двух групп по количественному показателю выполнялось с помощью U-критерия Манна-Уитни. Сравнение процентных долей при анализе четырехпольных таблиц сопряженности выполнялось с помощью критерия хи-квадрат (χ2) Пирсона (при значениях ожидаемого явления более 10) и с помощью точного критерия Фишера (при менее 10). Сравнение процентных долей при анализе многопольных таблиц сопряженности выполнялось с помощью критерия χ2 Пирсона. Направление и теснота корреляционной связи между двумя количественными показателями оценивались с помощью коэффициента ранговой корреляции Спирмена. Построение прогностической модели вероятности определенного исхода выполнялось при помощи метода логистической регрессии. Мерой определенности, указывающей на ту часть дисперсии, которая может быть объяснена с помощью логистической регрессии, служил коэффициент R² Найджелкерка. Статистически значимыми считались изменения при вероятности ошибочного принятия нулевой гипотезы р<0,05. Предполагалось включить в исследование 390–400 респондентов, что соответствует критериям репрезентативности выборки по методике определения объема выборки К.А. Отдельновой и др. [18].

## Этическая экспертиза

Исследование проводилось с письменного согласия волонтеров и в соответствии с требованиями Хельсинской Декларации Всемирной Медицинской Ассоциации об этических принципах проведения медицинских исследований (2000 г.), оно было одобрено этическими комитетами (протоколы заседаний этических комитетов Института физиологии природных адаптаций УрО РАН и ФГБУН ФИЦКИА УрО РАН от 2.02.2009, 4.02.2013, 9.11.2016).

## Результаты

Демографические данные и ИМТ (табл. 1)

Общее количество обследованных лиц составило 397 человек; из них 139 человек — жители НАО, 35 — Мезенского района АО и 223 волонтера из ЯНАО; 377 (95%) респондентов идентифицировали себя, согласно анкетным данным, как ненцы, 20 (5%) — как коми.

Группа КА составила 89 (22,4%) человек и поровну представлена мужчинами и женщинами. Группа ОА — 308 (77,6%), из них 239 (77,6 %) женщин. Группы не различались по возрасту: его медиана составила 43 года. Выборка была разделена на подгруппы по возрасту согласно классификации АПН СССР (Москва, 1965); статистической разницы между возрастными подгруппами также не выявлено. Медиана ИМТ 26,69 кг/м2 отвечала критерию избыточной массы тела (ИзМТ, ВОЗ, 1997 г.). При оценке показателя в зависимости от ОЖ статистически значимые различия не выявлены (p=0,669). Тенденциально (р=0,091) в группе КА отмечен более высокий процент лиц с ИзМТ, отсутствие случаев дефицита массы тела и меньше респондентов с ожирением по сравнению с ОА (табл. 1).

**Table:** Таблица 1. Гендерные различия, анализ возраста и ИМТ в зависимости от образа жизни коренных жителей Крайнего Севера Примечание: * — различия показателей статистически значимы (p<0,05). Категориальные данные представлены — абс. (%; 95% ДИ).

Показатели	Образ жизни	р
Кочующие	Оседлые
n=89 (22,4 %)	n=308 (77,6 %)	<0,001*
Пол	Муж.	44(49,4; 38,7–60,2)	69(22,4; 17,9–27,5)	<0,001*
Жен.	45(50,6; 39,1–61,3)	239(77,6; 72,5–82,1)
Возраст, летMe [Q₁-Q₃], мин–мах	42 [ 31–50]22–59	43 [ 33–50]22–60	0,372
Возраст, шкала	22–35 лет	35(39,3; 29,1–50,3)	105(34,1; 28,8–39,7)	0,661
36–45 лет	20(22,5; 14,3–32,6)	75(24,4; 19,7–29,5)
46–60 лет	34(38,2; 28,1–49,1)	128(41,6; 36,0–47,3)
ИМТ, кг/м2Me [Q₁-Q₃], мин–мах	26,56[ 23,74–29,48]18,73–39,76	26,88[ 23,23–30,83]15,62–44,24	0,669
ИМТ,шкала	<18,5 кг/м2	0 (0,0)	10(3,2; 1,6–5,9)	0,091
18,5–24,9 кг/м2	32(36,0; 26,1–46,8)	110(35,7; 30,4–41,3)
25–29,9 кг/м2	37(41,6; 31,2–52,5)	96(31,2; 26,0–36,7)
≥30 кг/м2	20(22,5; 14,3–32,6)	92(29,9; 24,8–35,3)

Инсулинемия и гликемия (табл. 2)

Медиана Инс 7,67 [ 4,37–12,78] мкЕд/мл в выборке в целом соответствовала низко-нормальным значениям. В группе КА уровень Инс был значимо (р=0,006) ниже, чем в группе ОА (табл. 2). Анализ случаев с показателем вне пределов референсных значений (т.е. менее 2,0 и более 25 мкЕд/мл) не выявил статистически значимых различий между группами.

Медиана Глю 4,61 [ 4,13–5,15] ммоль/л в исследуемой популяции соответствовала среднему значению референса. При сравнении Глю в зависимости от ОЖ статистически значимых различий не установлено. В группе КА отмечен более высокий процент лиц с эугликемией за счет отсутствия случаев, соответствующих критериям недиабетической гипогликемии (<3 ммоль/л) и сахарному диабету (≥7,0 ммоль/л), но статистическая значимость по данным различиям не достигнута (табл. 2).

Корреляционный анализ не выявил статистически значимой связи между Инс и Глю в группе КА (ρ=0,194; р=0,069). В группе ОА была установлена умеренной тесноты прямая связь (ρ=0,385; р<0,001). На рис. 1 продемонстрировано отсутствие линейной зависимости уровня Инс от гликемии в группе КА и наличие в ОА.

**Table:** Таблица 2. Уровни инсулина и глюкозы в зависимости от образа жизни коренных жителей Крайнего Севера Примечание: * — различия показателей статистически значимы (p<0,05). Категориальные данные представлены — абс. (%; 95% ДИ). ** Показатель представлен для понимания количества замен при расчете HOMA1-%β, с заменой***, см. табл. 3. *** Критерий метаболического синдрома (AHA/NHBLI, 2009 г.).

Показатели	Образ жизни	р
Кочующие	Оседлые
n=89 (22,4 %)	n=308 (77,6%)
Уровень инсулина и случаи гипо- и гиперинсулинемии
Инсулин, мкЕд/млMe [Q₁-Q₃] мин–мах	6,04[ 3,49–11,80]1,5–33,22	8,30[ 4,59–13,33]1,2–54,59	0,006*
Гипоинсулинемия<2,1 мкЕд/мл	5(5,6; 2,6–14,4)	9(2,9; 1,2–5,5)	0,255
Гиперинсулинемия>25 мкЕд/мл	4(4,5; 1,9–12,9)	22(7,1; 5,8–12,7)	0,374
Уровень гликемии и случаи нарушений углеводного обмена
Глюкоза, ммоль/лMe [Q₁-Q₃] мин–мах	4,62[ 4,22–5,04]3,09–6,27	4,61[ 4,11–5,16]2,71–10,24	0,845
Гипогликемия< 3,0 ммоль/л	0	6(1,9; 0,6–4,0)	0,236
Эугликемия≥ 3,0 <6,1 ммоль/л	86(96,6; 90,3 – 99,3)	279(90,6; 86,1–93,4)
НТГ≥ 6,1<7,0 ммоль/л	3(3,4; 0,7–9,7)	18(5,8; 3,8–9,8)
СД≥ 7,0 ммоль/л	0	5(1,6; 0,6–4,0)
≤ 3,5 ммоль/л**	4(4,5;1,2–11,1)	22(7,1; 4,8–11,1)	0,374
≥ 5,6 ммоль/л***	12(13,5; 7,2–22,4)	44(14,3; 10,6–18,7)	0,848

**Figure:**
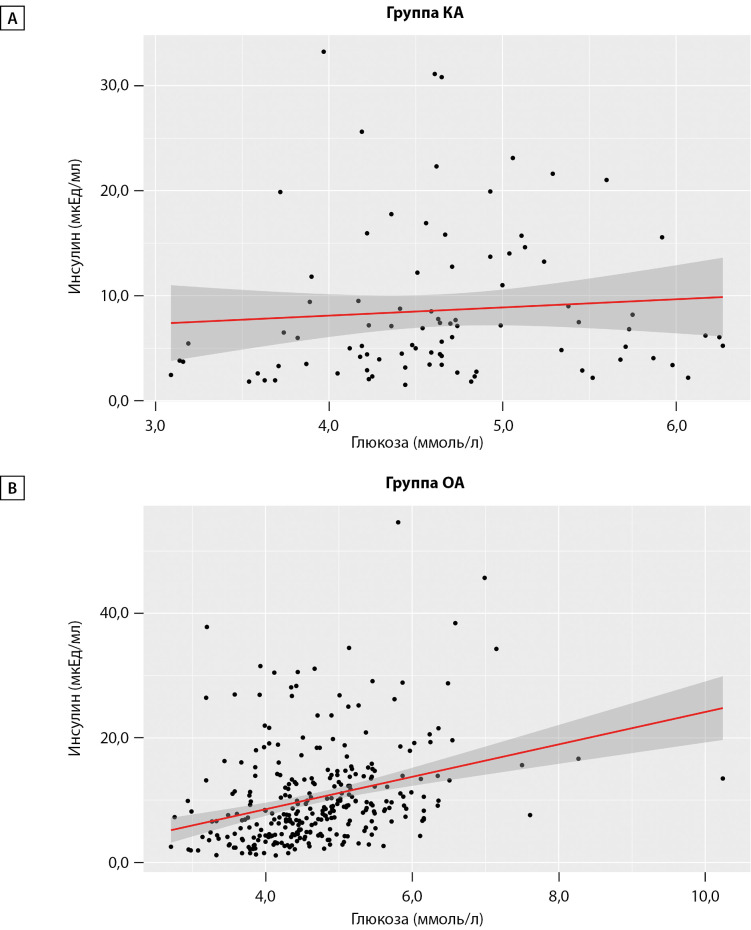
Рисунок 1. Графики, демонстрирующие отсутствие (А) и наличие (В) линейной зависимости инсулина от глюкозы у аборигенов с разным образом жизни.

Инсулинорезистентность и секреторная активность β-клетки (табл. 3, рис. 2)

Величина индекса IR-HOMA в эпидемиологических исследованиях как правило описывается значениями 75‰–90‰. Анализ IR-HOMA в зависимости от ОЖ показал меньшие значения 75‰–90‰ индекса у КА — 2, 44 и 4,38 усл.ед. в сравнении с 2,82 и 4,98 у ОА, p=0,013. Пороговое значение, определяющее наличие инсулинорезистентности, — IR-HOMA >2 усл.ед. [14] у КА соответствовало 72‰, у ОА — 55‰. Шансы наличия IR-HOMA>2 усл.ед. в группе ОА были в 2,17 (95% ДИ: 1,29–3,69) раза выше по сравнению с группой КА, р=0,012.

Нами была проведена скорректированная оценка ОШ для выбранных предикторов индекса «IR-HOMA>2» посредствам метода логистической регрессии. Многофакторная бинарная логистическая Модель (1), включившая в себя ОЖ, пол, шкалы ИМТ и возраста, была статистически значимой (p=0,026), и независимое влияние ОЖ на показатель «IR-HOMA>2» сохранилось с шансами наличия в 1,77 раза ниже у КА в сравнении с ОА. Исходя из значения коэффициента детерминации R2 модель объяснила 5,3% наблюдаемой дисперсии значения индекса «IRHOMA>2 усл.ед.», рис. 2 (А).

Далее была оценена Fβ. Медиана индекса HOMA1-%β, рассчитанная, согласно условиям использования индекса [16], только у лиц с уровнем Глю≥3,5 ммоль/л (n=371), соответствовала высоким значениям, без значимых различий между группами (p=0,163). Нами был рассчитан индекс HOMA%β «с заменой»: у 26 участников (4 КА и 22 ОА), чьи показатели Глю были ниже или равны 3,5 ммоль/л. При расчете индекса в формулу вводили значение Глю, равное 3,51 ммоль/л. Данная манипуляция позволила увидеть тенденцию (р=0,096) к более высоким значениям Fβ в группе ОА (табл. 3).

Пороговое значение, определяющее наличие гипофункции β-клетки (гипо-Fβ), составляет менее 48,9% по величине индекса HOMA1-%β [16]. Оценка доли респондентов по этому признаку показала существенные различия между группами: 16,9% у КА и 4,9% у ОА, p<0,001, т.е. шансы наличия респондентов с низкой секреторной активностью β-клетки в группе КА оказались в 4 раза выше по сравнению с группой ОА, ОШ=0,25; 95% ДИ: 0,12–0,54, p<0,001.

С помощью метода логистической регрессии, по аналогии с анализом показателя «IR-HOMA>2», была проведена скорректированная оценка ОШ для тех же предикторов показателя «HOMA1-%β<48,9». Регрессионная модель (2) сохранила значимость (p=0,007) с шансами наличия гипо-Fβ в группе КА в 3,51 раза выше по сравнению с ОА. Модель объяснила 11,6% (R2=0,116) наблюдаемой дисперсии показателя «HOMA1-%β <48,9». На рис. 2 (В) сопоставлены значения скорректированного ОШ с 95% ДИ для изучаемых факторов Модели 2.

**Table:** Таблица 3. Уровни инсулинорезистентности и секреторной активности β-клетки в зависимости от образа жизни коренных жителей Крайнего Севера Примечание: * — различия показателей статистически значимы (p<0,05). Категориальные данные представлены — абс. (%; 95% ДИ). Индекс HOMA1-%β с исключением**у лиц с гликемией менее 3,51 ммоль/л. Индекс HOMA1-%β, с заменой*** (все значения гликемии ≤3,5 заменены на 3,51 ммоль/л).

Показатели	Категория — образ жизни	р
Кочующие	Оседлые
n=89 (22,4 %)	n=308 (77,6 %)
Чувствительность к инсулину
IR-HOMA, усл. ед.	1,30 [ 0,70–2,44]0,28–6,37	1,82 [ 0,95–2,82]0,17–14,19	0,013*
Инсулинорезистентность
IR-HOMA ≥2 усл. ед.	26(29,2; 20,1–39,8)	136(44,2; 38,5–49,9)	0,012*
IR-HOMA ≥2 усл. ед.,мужчины	5(11,4; 31,7–62,1)	34(49,3; 37,0–61,6)	<0,001*
IR-HOMA ≥2 усл. ед.,женщины	21(46,7; 31,7–62,1)	102(42,7; 36,3–49,2)	0,620
IR-HOMA ≥2 усл. ед.,при гликемии ≥5,6 ммоль/л	3(25,0; 5,5–57,2)	37(84,1; 69,9–93,4)	<0,001*
Секреторная активность β-клетки
HOMA1-%β с исключением**	n=85	n=286	0,163
128,51[ 67,02–241,34]16,96–1805	144,39[ 93,37–236,48]25,02–6737
HOMA1-%β,с заменой***	n=89	n=308	0,096
135,11[ 68,44–262,98]16,96–10880	150,44[ 96,94–296,79]25,02–75580
Гипофункция β-клетки
HOMA1-%β <48,9%	15(16,9; 9,8–26,3)	15(4,9; 2,8–7,9)	<0,001*
HOMA1-%β <48,9%, мужчины	12(27,3; 15,0–42,8)	3(4,3; 0,9–12,2)	<0,001*
HOMA1-%β <48,9%, женщины	3(6,7; 1,4–18,3)	12(5,0; 2,6–8,6)	0,651
HOMA1-%β <48,9%,при гликемии ≥5,6 ммоль/л	8(66,7; 34,9–90,1)	4(9,1; 2,5–21,7)	<0,001*

**Figure:**
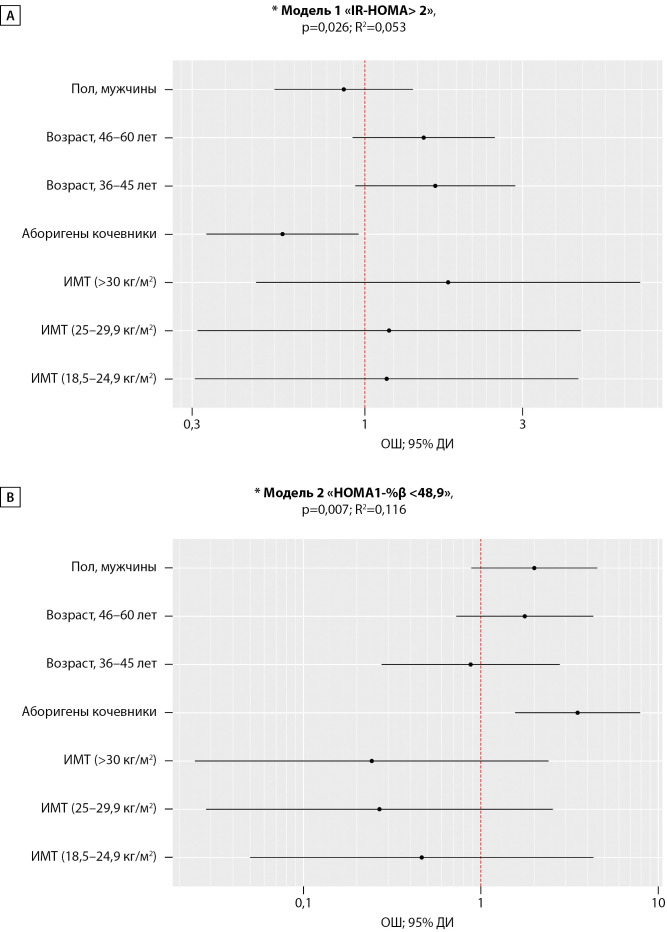
Рисунок 2. Оценка отношения шансов для предикторов (образ жизни, пол, возраст, ИМТ) показателя «IR-HOMA> 2» (А) и «HOMA1-%β <48,9» (В) у аборигенов Арктики. Примечание: *Основные характеристики регрессионных моделей, р<0,05 — статистическая значимость и коэффициент детерминации (R2). А — «ОЖ, кочевники», ОШ = 0,56; 95% ДИ: 0,33 – 0,96, р=0,034. В — «ОЖ, кочевники», ОШ = 3,51; 95% ДИ: 1,56 – 7,92, р=0,002.

Инсулинорезистентность и секреторная активность β-клетки у мужчин (табл. 3, рис. 3)

И в Модели 1, и в Модели 2 отмечалось однонаправленное с предиктором «ОЖ» влияние категории «пол, мужской» на долю лиц с IR и гипо-Fβ. Поэтому далее мы оценили эти параметры у мужчин (n=113).

Медиана, 75‰ и 90‰ индекса IR-HOMA в группе мужчин кочевников составили 0,98–1,53–2,68 усл.ед. и были ниже в сравнении с 1,98–2,76–3,69 усл.ед. в группе ОА мужчин, р<0,001.

Регрессионная Модель 3 (n=113), выполненная аналогично Модели 1, но в отношении подгруппы мужчин с целью оценки ОШ изучаемых предикторов на наличие «IR-HOMA> 2», была статистически значимой (p<0,001) и исходя из значения R2 объяснила 27,2% его дисперсии (рис. 3), т.о. шансы наличия IR-HOMA>2 усл.ед. у мужчин КА были в 10 раз ниже, чем у оседлых, ОШ=0,10; 95% ДИ: 0,03–0,31, р<0,001.

Ме [IQR] «HOMA1-%β» тенденциально ниже была в группе мужчин кочевников и составила 99,67 [ 46,41–192,99] % и 130,00 [ 91,26–196,23] % в группе оседлых, р=0,054. Логистическая регрессия (Модель 4, рис. 3) характеризует шансы наличия у мужчин гипоFβ, объяснила 22% (R2=0,220, =0,014) наблюдаемой дисперсии индекса «HOMA1-%β <48,9». Скорректированное ОШ наличия гипо-Fβ у мужчин оленеводов оказалось в 8,3 раза выше относительно мужчин оседлого образа жизни (95% ДИ: 2,15–32,17, р=0,002). При этом различий в величине ИМТ между КА и ОА выявлено не было: 26,24±3,78 и 26,56±4,10 кг/м2, р=0,672 соответственно.

Ме [IQR] «HOMA1-%β» тенденциально ниже была в группе мужчин кочевников и составила 99,67 [ 46,41–192,99]%, а в группе оседлых 130,00 [ 91,26–196,23]%, р=0,054. Логистическая регрессия (Модель 4, рис. 3) характеризует шансы наличия гипоFβ у аборигенов мужчин, оказавшиеся в 8,3 раза выше в группе КА по сравнению с ОА (95% ДИ: 2,15–32,17, р=0,002). При этом различий в величине ИМТ между КА и ОА не наблюдалось: 26,24±3,78 и 26,56±4,10 кг/м2, р=0,672 соответственно. Модель 4 объяснила 22% (R2=0,220, =0,014) наблюдаемой дисперсии индекса «HOMA1-%β <48,9».

**Figure:**
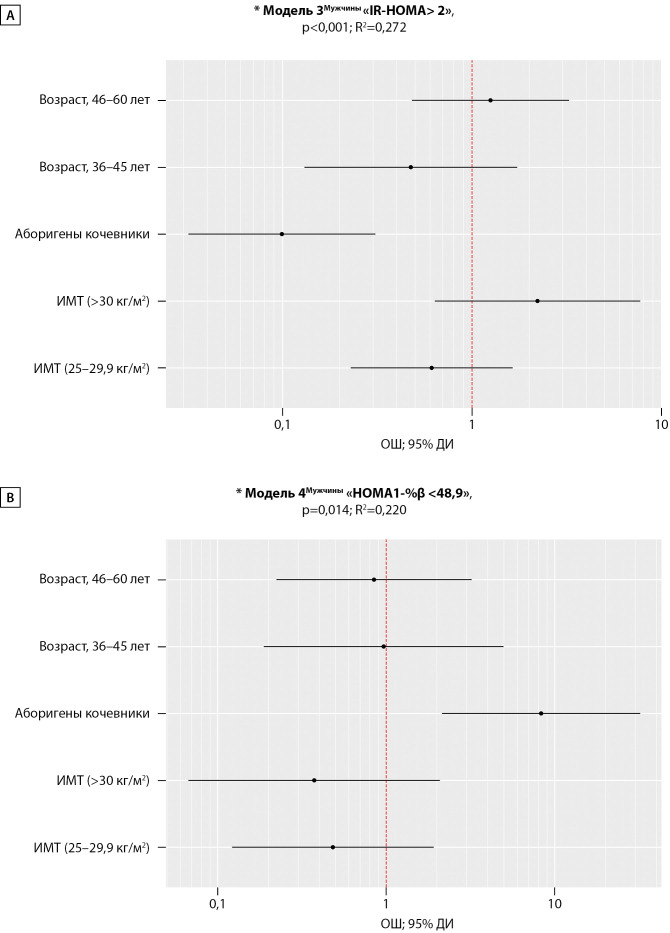
Рисунок 3. Оценка отношения шансов для предикторов (образ жизни, пол, возраст, ИМТ) показателя «IR-HOMA> 2» (А) и «HOMA1-%β <48,9» (В) среди мужчин. Примечание: *Основные характеристики регрессионных моделей, р<0,05 — статистическая значимость и коэффициент детерминации. А — «ОЖ, кочевники», ОШ; 95% ДИ (0,10; 0,03 – 0,31), р<0,001. B — «ОЖ, кочевники», ОШ; 95% ДИ (8,3; 2,15 – 32,17), р=0,002.

Инсулинорезистентность и секреторная активность β-клетки у женщин (рис. 4)

Величина индекса IR-HOMA у коренных жительниц Арктики не различалась относительно ОЖ: 1,76 [ 0,88–3,28] усл.ед. в группе КА и 1,68 [ 0,90–2,88] усл.ед. в группе ОА, р=0,727. Fβ была высокая и также не различалась у КА относительно ОА; индекс HOMA1-%β составил 188,65 [ 97,57–412,79]% и 156,43 [ 98,47–365,51]%, р=0,697 соответственно.

Регрессионные Модели 5 и 6 (n=284), построенные с целью оценки ОШ изучаемых предикторов на «IR-HOMA>2» и «HOMA1-%β <48,9», не были статистически значимыми, (рис. 4). Значимое влияние на величину «IR-HOMA>2» у женщин имелось только у категории «возраст, шкала» — ОШ; 95%ДИ (2,36; 1,22–4,57), р=0,010. Скорректированные шансы наличия у них IR оказались в 2,4 раза выше в возрасте 35–45 лет вне зависимости от OЖ. Влияния изучаемых предикторов на наличие «HOMA1-%β <48,9» у женщин не выявлено.

**Figure:**
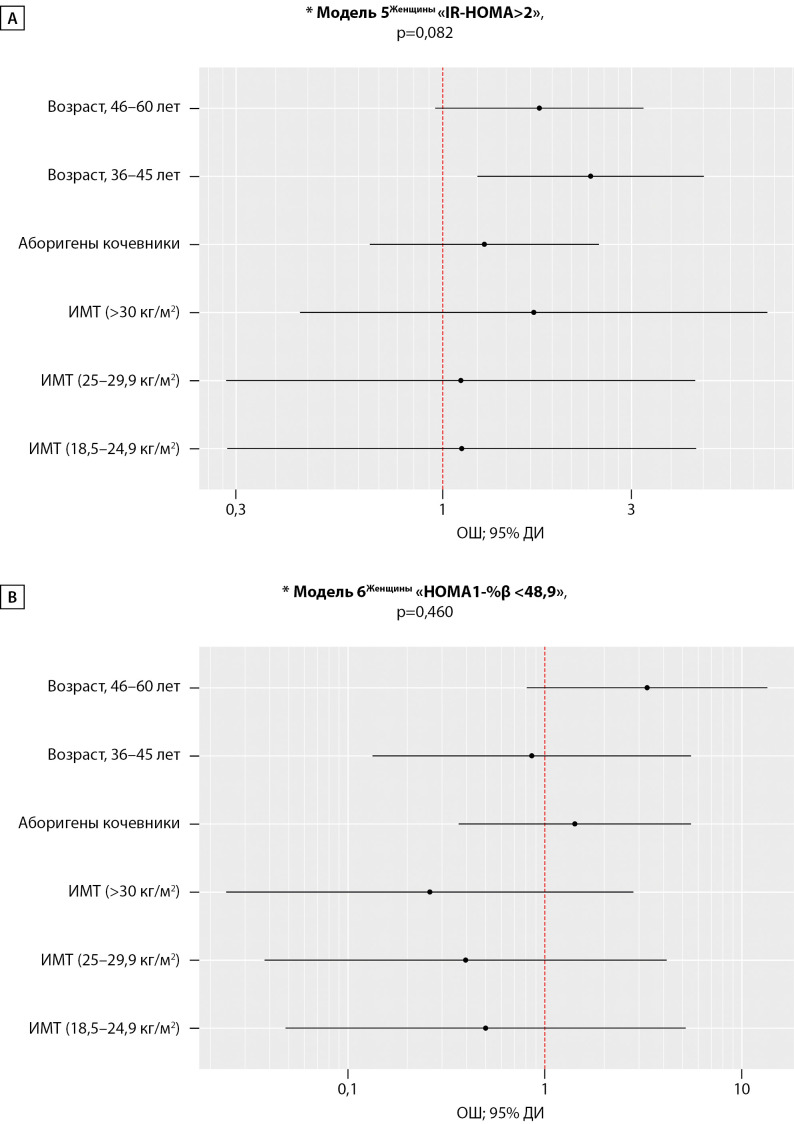
Рисунок 4. Оценка отношения шансов для предикторов (образ жизни, пол, возраст, ИМТ) показателя «IR-HOMA> 2» (А) и «HOMA1-%β <48,9» (В) среди женщин. Примечание: *Основные характеристики регрессионных моделей, р<0,05 — статистическая значимость и коэффициент детерминации. А — «Возраст, 35-45 лет»; ОШ; 95% ДИ (2,37; 1,22 – 4,57), р=0,010.

Инсулинорезистентность и секреторная активность β-клетки при гликемии ≥5,6 ммоль/л (рис. 5)

Последним этапом работы был анализ случаев «IR-HOMA>2» и «HOMA1-%β <48,9» при гликемии ≥5,6 ммоль/л (n=56) как одного из критериев метаболического синдрома (AHA/NHBLI, 2009 г.). Частота случаев Глю≥5,6 ммоль/л оказалась сходной с учетом численности изучаемых популяций: у КА — 12 (13,5%), в группе ОА — 44 (14,3%), табл. 2. Медиана индекса IR-HOMA у КА с Глю≥5,6 ммоль/л составила 1,57 [ 1,04–1,82] усл.ед.; у ОА значимо выше — 3,02 [ 2,27–5,70], р<0,001; как и величина индекса HOMA%β — 45,15 [ 35,36–63,92]% у КА в сравнении с ОА — 88,12 [ 67,96–146,34] %, р=0,002. Таким образом, шансы наличия IR у КА с уровнем Глю≥5,6 ммоль/л оказались в 15,86 раза ниже по сравнению с группой ОА (95% ДИ: 3,41–73,69; p<0,001). Тогда как шансы наличия гипоFβ у КА с аналогичной Глю были в 20 раз выше по сравнению с ОА (ОШ=0,05; 95% ДИ: 0,01–0,24; p<0,001).

При сравнении ИМТ (M±SD (95% ДИ)) у этих лиц выявлены значимые различия (р=0,006). В группе КА ИМТ оказался существенно ниже, чем у ОА: 25,16±2,86 (23,34–26,97) и 29,57±5,13 (28,01–31,13) кг/м2, р=0,006. У КА не было случаев ожирения у лиц с Глю≥5,6 ммоль/л, и шансы его наличия при этих условиях были в 27,33 раза ниже по сравнению ОА, (95% ДИ: 1,52–490, р<0,001), рис. 5.

**Figure:**
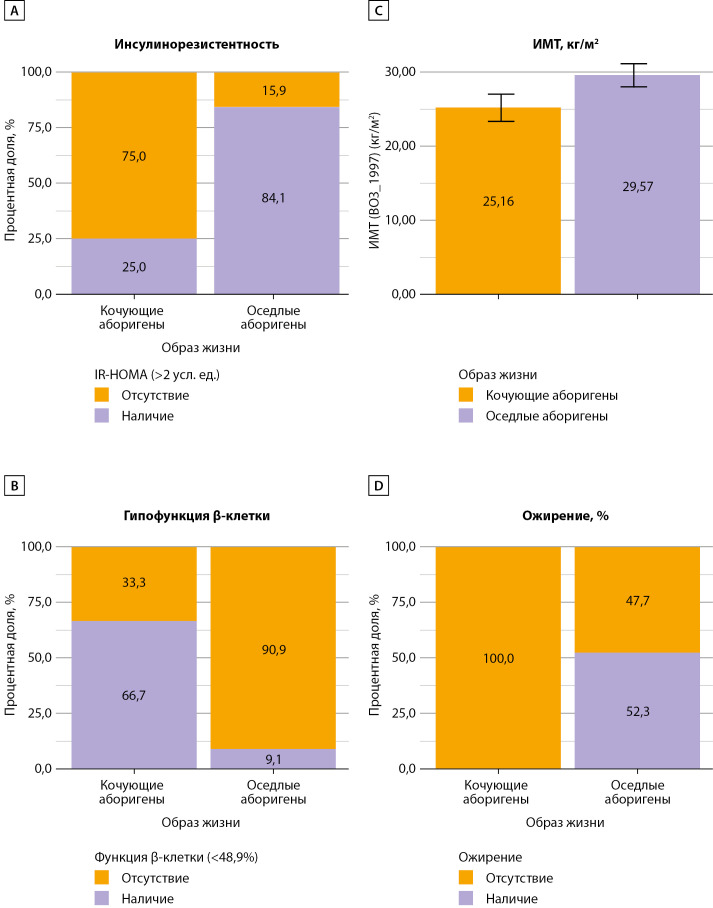
Рисунок 5. Соотношение случаев инсулинорезистентности и гипофункции β-клетки при гликемии ≥ 5,6 ммоль/л у кочующих и оседлых аборигенов с анализом ИМТ. Примечание: А, В, С — данные представлены в %, D — M±SD. А — «IR-HOMA> 2» усл. ед. выявлена у 3-х КА (25%) и у 37 (84,1%) ОА, p<0,001; В — «HOMA1-%β<48,9», % – у 8 (66,7%) КА и у 4 (9,1 %) ОА, р<0,001; С — ИМТ, кг/м2, р=0,006; D — Отсутствие ожирения у 12 КА, наличие у 23(52,3%) из 44 ОА, %, p<0,001.

## Обсуждение

## Репрезентативность выборок

Выборка представлена преимущественно ненцами — древнейшей из ныне проживающих групп коренного населения Арктики. Ненцы считаются самым многочисленным малым народом России; срок обитания в Европейском Заполярье — несколько тысячелетий. Коми (зыряне) не относятся к малым народам, проживают на территории Арктики несколько столетий. Участники были отобраны случайным образом зимне-весенними экспедициями, составили 2,15% от численности, аналогичной по возрасту популяции коренного сельского оседлого и кочующего населения этих территорий (согласно Всероссийской переписи 2010 г.), что соответствует критериям репрезентативности выборки, трактуется как исследование повышенной точности, с величиной допускаемой ошибки 5% по методике определения объема выборки К.А. Отдельновой, В.И. Паниотто и N. Fox. [18]. Отсутствие равновесия по полу в группе ОА связано с нежеланием обследоваться мужчин ОА. Малочисленность КА обусловлена отдаленностью стоянок от пунктов обследования.

## Сопоставление с другими публикациями

Опубликовано много работ, посвященных особенностям метаболизма, в том числе исторически минимизированному углеводному обмену у коренных жителей Арктики. Данные об уровне гликемии разнятся [9][19][20], но, как правило, свидетельствуют о том, что до эпохи «метаболической пандемии» на фоне многовекового традиционного уклада жизни показатели соответствовали 1 и 2 квартилю референсных значений, т.е. низко-нормальным [6, 10]. Некоторые исследователи отмечали, что аборигенам свойственна тенденция к гипогликемии без ее клинических проявлений [10]. Мы также выявили 6 (1,5%) случаев недиабетической гипогликемии (<3,0 ммоль/л) и только среди ОА.

Более поздние публикации сообщают о нивелировании разницы в уровне глюкозы плазмы (сыворотки) у жителей Заполярья от среднеширотной нормы, появлении и росте в популяциях ожирения и СД [4][5][12][13][21][22]. Также отмечено, что имеется цикличная трансформация метаболических процессов как минимум в рамках годичного ритма экстремальных климатогеографических факторов Крайнего Севера [23]. Поэтому в нашем исследовании мы представляем результаты только зимне-весеннего периода.

Полученные нами данные об уровне Глю 4,6 ммоль/л по медиане, равнозначные у КА и ОА, свидетельствуют, что у половины выборки показатели гликемии соответствуют порогу (>4,44 ммоль/л), при котором предполагается физиологическая реакция организма в виде повышения секреции Инс, стимуляции захвата Глю тканями, усиления гликогенеза в печени и мышцах, липогенеза в липоцитах как защита от гипергликемии [24].

Вместе с тем мы обнаружили, что кочевники в меньшей степени наращивают уровень Инс по сравнению с ОА, и демонстрируют отсутствие линейной зависимости между уровнем Глю и Инс (рис. 1А). По данным литературы, для аборигенов Арктики, сохраняющих традиционный ОЖ, характерна способность осуществлять обменные процессы при пониженной нагрузке на β-клетки [25–27]. Исследования же последних лет, тем не менее, сообщают о росте инсулинемии у аборигенов Арктики [28][29], что согласуется и с нашими данными (рис. 1Б). В нашем исследовании гиперинсулинемия отмечена у 26 (6,5%) человек, без статистической разницы между группами. Для выборки в целом характерны невысокие показатели Инс, но на 27% статистически ниже уровень Инс оказался у кочевников — 6,04 [ 3,49–11,80] мкЕд/мл при идентичной с ОА гликемии, отсутствии различий в ИМТ и возрасте.

Повышение уровня Инс в популяциях жителей Севера сопряжено с ростом резистентности к Инс и ассоциированных состояниях/заболеваниях [4][28][29]. В нашей работе мы также отмечаем, что смена уклада жизни ассоциируется с потерей чувствительности к Инс.

Однако нам не встретилось работ, посвященных анализу чувствительности к Инс с одновременной оценкой секреторных возможностей β-клетки в популяциях аборигенов Арктики. Вместе с тем доказательства не только «сдержанной» работы инсулярного аппарата, но и эпизодов «секреторного бессилия» или транзиторной стресс-обусловленной гипофункции β-клетки у жителей Крайнего Севера известны давно.

Так, в 1928 г. Р. Heinbecker [9] описал гликемию, глюкозурию и кетонемию у 7 эскимосов Канады на фоне углеводной нагрузки при традиционном питании (n=4) и после 82-часового голодания (n=3). Им была дана характеристика пищевого рациона аборигенов, показавшая существенное превалирование белково-липидного компонента: 60% (280 г) — белок, 29% (135 г) — жир и 11% (54 г) — углеводы, т.е. 2551 ккал/сут. при средней массе тела женщин 50–60 кг, мужчин 60–70 кг. При этом уровни Глю натощак у обследованных оказались выше среднеширотных ~100–120 мг/дл (5,55–6,66 ммоль/л). В ходе нагрузки Глю (2 г/кг) при привычном пищевом поведении эскимосы демонстрировали высокую толерантность к углеводам с максимальным подъемом Глю до ~140 мг/дл (7,77 ммоль/л) на первом из 4 часов теста, без сопутствующей глюкозурии, с плавной нормализацией Глю до уровней натощак и ниже ~ 80–100 мг/дл (4,44–,55 ммоль/л), но не до гипогликемии. Данные аналогичной нагрузки, но после 82-часового голодания оказалась более интересными: эскимосы, в популяциях которых до 50-х годов прошлого столетия не было случаев заболевания СД, продемонстрировали 3-кратное повышение Глю на первых часах теста (~300 мг/дл или 16,65 ммоль/л) с сопутствующей глюкозурией, сохраняющейся до 10–43 часов и более 25-часовым шлейфом гипергликемии. Были изучены уровни кетонурии, азотемии и показатели основного обмена аборигенов. Ученый сделал выводы об исключительной адаптивной способности к полному окислению жиров, высокой чувствительности к углеводам при традиционном питании с утратой толерантности к углеводам на фоне голода; «мягкой» склонности к голодному кетозу и антикетогенных эффектах белкового рациона. Он показал, что метаболизм эскимосов в состоянии покоя на 33% выше, чем у жителей средних широт. Таким образом, в 1928 г. впервые был индуцирован (острой массивной нагрузкой углеводами в условиях голодного стресса) и продемонстрирован обратимый транзиторный СД у коренного жителя Арктики. Разумное «отступление» β-клетки не явилось «поражением», т.к. сдержало развитие компенсаторной гиперинсулинемии и инсулинорезистентности с целью сохранения возможности «легкого» окисления жиров, а главное — «трудного» глицеринового и белкового глюконеогенеза.

В дальнейшем Л.Е. Панин (1978 г.), исследователь Азиатской Арктики, автор концепции «полярного метаболического типа», описал «функциональный транзиторный, экологически обусловленный диабет напряжения» у жителей Крайнего Севера, отметив в целом популяционную склонность аборигенов к низко-нормальной гликемии [6][7]. Предпосылки к развитию подобного диабета с низкими концентрациями инсулина он объяснил, в первую очередь, контринсулярным действием высоких относительно средних широт уровней стероидов надпочечников и катехоламинов, обеспечивающих термогенез [6][7]. Во-вторых, он считал, что белок аполипопротеин В (апоВ), входящий в структуру ЛПНП и ЛПОНП, уровень которых, как правило, высок у аборигенов, подавляет синтез и секрецию инсулина [7]. Третьей причиной, противостоящей развитию гиперинсулинемии и способствующей выявлению диабета, по его мнению, явилось эпизодическое снижение реабсорбции глюкозы из первичной мочи [7]. Л.Е. Панин описал феномен транзиторной глюкозурии у жителей Крайнего Севера не только в состоянии легкой гипергликемии, но и при эугликемии, и даже при низко-нормальном уровне гликемии как адаптивный механизм, способствующий поддержанию гипоинсулинемии в периоды стресса и сезонных гормонально-метаболических перестроек, т.е. при необходимости торможения гликолиза и повышении потребности в утилизации жиров в качестве основного источника энергии, задолго до понимания роли ингибиторов натрий-глюкозных ко-транспортеров (SGLT2) в терапии СД и сердечной недостаточности. Можно предположить, что коренные жители Арктики, сохраняющие низко-нормальный уровень Инс, способны к модуляции порога реабсорбции Глю в почках как защите от гиперинсулинемии. Не исключено, что «особая вовлеченность» SGLT2 в энергетический баланс может быть дополнительным ресурсом, обеспечивающим низкие кардиометаболические риски в условиях сохранения традиционного уклада жизни.

В нашем исследовании совместный анализ индексов HOMA-IR и HOMA1-%β выявил случаи «сдерживания» секреторной активности β-клетки преимущественно в группе КА мужчин. Так, 71% КА и 89% мужчин кочевников продемонстрировали отсутствие инсулинорезистентности, при этом у 17% КА и у 27% мужчин КА выявлена гипофункция β-клетки. В группе ОА, наоборот, нарастало число лиц с инсулинорезистентностью — 44% (49% мужчин, 43% женщин) при сохранении секреторной активности β-клетки у 95% респондентов вне зависимости от пола. Образ жизни стал независимым предиктором, влияющим на число случаев гипофункции β-клетки и резистентности к инсулину.

Известно, что метаболизм коренного жителя Крайнего Севера филогенетически настроен на эффективное окисление ЖК. Этому способствует приверженность к традиционному питанию и необходимость адаптации к холоду [30]. Жирные кислоты не только активно окисляются в бурой и бежевой жировой ткани, но и в мышечной. При высокой доступности ЖК они конкурируют с глюкозой и ингибируют ее утилизацию в мышцах (Randle P.J., 1964 г.). Согласно первичной гипотезе Рэндла, увеличение метаболизма жирных кислот приводит к увеличению содержания интрамитохондриального КоА, снижению соотношения (НАДН)/НАД+ с последующим ингибированием пируватдегидрогеназы. Внутриклеточное увеличение концентрации цитрата в митохондриях (и цитозоле) аллостерически ингибирует фосфофруктокиназу, контролирующую гликолиз. Последующее накопление глюкозо-6-фосфата ингибирует активность гексокиназы II, что приводит к увеличению содержания Глю в клетке, «запиранию» и снижению поглощения Глю, которая в условиях преимущественного окисления жиров может без проблем проникать в кровоток и расходоваться мозгом. Ключевым требованием этого процесса являлось низкое содержание инсулина [31]. Таким образом, Рендл описал механизм повышения уровня гликемии в крови при низком уровне инсулина в условиях преимущественного окисления жиров in vitro, а Р. Heinbecker в начале прошлого века при обследовании инуитов Канады, выявив у них на исключительно белково-жировом питании относительную гипергликемию натощак, подтвердил это in vivo [17].

Возможно, одной из причин разночтений в уровне гликемии у коренных жителей Арктики прошлого столетия может быть разница в типе, режиме приема и количестве углеводов, постепенно проникавших в рацион, и, таким образом, более отчетливая конкуренция между глюкозой и жирными кислотами за окисление в мышечной ткани, чувствительной к транзиторному повышению инсулина.

При перманентном повышении количества инсулина (модель менее чувствительного к инсулину оседлого аборигена), в ситуации доступности обилия жирных кислот, как с пищей, так и за счет абдоминального липолиза при метаболическом синдроме, а также снижении трат на поддержание термогенеза [30] (как сократительного, так и несократительного), классический цикл Рэндла нарушается. Основной эффект жирных кислот в данном случае состоит в снижении транспорта глюкозы в мышечную в клетку, что доказано уменьшением скорости накопления внутриклеточной глюкозы и гликогена с помощью 13С и 31Р ядерно-магнитной-резонансной спектроскопии [31]. В эксперименте у здоровых лиц острое увеличение СЖК, достигнутое с помощью инфузии эмульсии триглицеридов и гепарина натрия (для активации липопротеинлипазы), приводило к падению внутриклеточных концентраций глюкозы и глюкозо-6-фосфата, предшествующих падению накопления гликогена [31]. Эти результаты не столько оспаривают гипотезу Рэндла, которая предсказывает увеличение концентраций внутриклеточного глюкозо-6-фосфата как основу «снижения» чувствительности к инсулину (повышения гликемии), сколько показывают, что в условиях иного гормонального обеспечения (гиперинсулинемии) и пищевого поведения аналогичная концентрация глюкозы в крови достигается в ходе разных метаболических механизмов. Подобное снижение транспорта глюкозы наблюдают у пациентов с СД2 и у части потомков больных СД2 с нормогликемией и инсулинорезистентностью [31].

В нашем исследовании мы показали два механизма развития гипергликемии в зависимости от образа жизни: у кочевников, преимущественно мужчин (т.е. лиц с большей относительно женщин мышечной массой, и длительнее в сравнение с ОА подвергающихся холодовому воздействию [30]) — за счет торможения секреторной активности β-клетки, а у оседлых — вследствие нарастания резистентности к инсулину. Мы не можем высказываться о стойкости этих изменений в силу дизайна исследования. Однако предположить, что в ряде случаев, на фоне стресса, в том числе пищевого, инфекции и прочее, транзиторный физиологический инсулинопенический синдром может трансформироваться в перманентный, возможно.

Нами также показано, что у женщин образ жизни не стал предиктором ни низкой секреторной активности β-клетки, ни резистентности к инсулину. Зависимость отмечена от возраста, что согласуется с данными популяционных исследований [32]. Интересным, на наш взгляд, оказался факт, что 35–46-летние женщины более резистентны к инсулину, чем представительницы старшего поколения аборигенов, вне зависимости от образа жизни, что может косвенно свидетельствовать о былом метаболическом благополучии жителей Крайнего Севера и согласуется с мировыми тенденциями «омоложения» ожирения и метаболического синдрома [33].

## Клиническая значимость результатов

Заключается в демонстрации двух разных механизмов формирования нарушений углеводного обмена у коренного населения Крайнего Севера. Так, у аборигенов оленеводов мужчин чаще развивается «транзиторный инсулинопенический диабет напряжения», его следует изучать в аспекте адаптации — дезадаптации и возможной трансформации в СД 1 типа; дифференцировать с СД1 (LADA). У оседлых аборигенов гипергликемия, как правило, обусловлена повышением резистентности к инсулину и требует мер по профилактике метаболического синдрома и СД2.

## Ограничения исследования

Ограничением исследования была неравномерность выборки с существенным преобладанием женщин оседлого образа жизни; поэтапный сбор материала — несколькими экспедициями в течение 10 лет, отсутствие возможности использовать для анализа компьютерною версию оценки Fβ и IR модель — HOMA2 (www.OCDEM.ox.ac.uk ), недостаточная мощность выборки для выявления случаев интолерантности к глюкозе и СД.

## Направления дальнейших исследований

Планируем оценить содержание проинсулина, С-пептида, лактата, пирувата, спектра жирных кислот в этой популяции и оценить их относительно характера питания аборигенов на современном этапе.

## Заключение

Сохранение кочевого образа жизни способствует удержанию более низкой концентрации инсулина. Сходные уровни глюкозы в группах обусловлены у КА, преимущественно мужчин, высокой долей лиц с низкой секреторной активностью β-клетки, тогда как ОА более резистентны к инсулину. Анализ случаев с гликемией ≥5,6 ммоль/л подтвердил генез гипергликемии у КА за счет гипофункции β-клетки с отсутствием случаев ожирения; у ОА — вследствие снижения чувствительности к инсулину, ассоциированным с развитием ожирения.

## Дополнительная информация

Источники финансирования. Исследование выполнено в соответствии с планом ФНИР по теме «Эндокринное обеспечение и характер питания в формировании адаптивных изменений в липидном обмене у различных групп населения Арктики на современном этапе» (гос. задание ФГБУН ФИЦКИА УрО РАН; рег. №НИОКТР 122011800399-2).

Конфликт интересов. Авторы декларируют отсутствие явных и потенциальных конфликтов интересов, связанных с содержанием настоящей статьи.

Участие авторов. Стрелкова А.В. — концепция, дизайн, анализ и интерпретация результатов, написание статьи; Бичкаева Ф.А. — получение, анализ данных, внесение в рукопись существенной правки с целью повышения научной ценности статьи; Власова О.С. — интерпретация данных, внесение в рукопись существенной правки с целью повышения научной ценности статьи; Нестерова Е.В.— получение данных; Шенгоф Б.А. — получение данных; Грецкая Т.Б. — получение данных. Все авторы одобрили финальную версию статьи перед публикацией, выразили согласие нести ответственность за все аспекты работы, подразумевающую надлежащее изучение и решение вопросов, связанных с точностью или добросовестностью любой части работы.
